# Treatment outcomes of multi-drug-resistant and rifampicin-resistant tuberculosis with and without isolation of nontuberculous mycobacteria between 2018–2021: A retrospective cohort study in Ghana

**DOI:** 10.1371/journal.pntd.0013204

**Published:** 2025-07-02

**Authors:** Elizabeth Tabitha Abbew, Roger Laryea, Ama Owusuaa Kwakye, Yaw Adusi Poku, Dorcas Obiri-Yeboah, Lutgarde Lynen, Tom Decroo, Leen Rigouts, Natalie Lorent

**Affiliations:** 1 Department of Clinical Sciences, Institute of Tropical Medicine, Antwerp, Belgium; 2 Department of Internal Medicine, Cape Coast Teaching Hospital, Cape Coast, Ghana; 3 Department of Biomedical Sciences, University of Antwerp, Belgium; 4 Eastern Regional Hospital, Koforidua, Ghana; 5 National Tuberculosis Control Programme, Accra, Ghana; 6 Department of Microbiology and Immunology, School of Medical Sciences, University of Cape Coast, Cape Coast, Ghana; 7 Department of Biomedical Sciences, Institute of Tropical Medicine, Antwerp, Belgium; 8 Department of Respiratory Diseases, University Hospital Leuven, Leuven, Belgium; 9 Department of Chronic Diseases, Metabolism and Aging, BREATHE Laboratory, Katholieke Universiteit Leuven, Leuven, Belgium; Yale University School of Medicine, UNITED STATES OF AMERICA

## Abstract

Multi-drug-resistant and rifampicin-resistant tuberculosis (MDR/RR-TB) pose an urgent health threat in Ghana. Despite ongoing interventions, the outcomes for MDR/RR-TB in Ghana have remained suboptimal over recent years. During this period, there has been an increasing detection of nontuberculous mycobacteria (NTM) in mycobacterial cultures. We sought to examine if the isolation of NTM could be a factor contributing to unfavourable MDR/RR-TB treatment outcomes. We also estimated predictors of NTM isolation, including using the short-course injectable-containing regimen (SCI) versus the all-oral bedaquiline (SCO) regimen and other covariates. This retrospective cohort study analysed MDR/RR-TB patients in Ghana from 2018 to 2021 across four regions. Demographic, clinical, and diagnostic data were collected under the National Tuberculosis Control Program framework. Mycobacterial smears and cultures were used to monitor treatment response, with further identification of NTM using line probe assays and Sanger sequencing. Multivariable logistic regression models evaluated predictors of NTM isolation and having an unfavourable outcome. Of 427 identified MDR/RR-TB patients, 380 were included for analysis: 76.3% were male, the mean age was 43.9 years, and 18.9% were people living with HIV. NTM were isolated in 7.1% of cases, primarily *Mycobacterium intracellulare* and *M. fortuitum*, with higher odds of isolation in individuals from the Eastern Region (aOR:14.18, 95% CI: 3.95-50.92). Overall, 67.9% achieved favourable outcomes: 71.4% (185/259) in those on the SCO versus 60.3% (73/121) on the SCI regimen. People living with HIV (aOR 14.18, 95% CI: 3.95-50.92) had an increased odds of having an unfavourable outcome. NTM isolation was not associated with unfavourable outcomes. Our study results suggest that although NTM isolation may occur during the course of MDR/RR-TB treatment, it does not affect MDR/RR-TB treatment outcome. Future research should further explore the implications of NTM co-infection on longer-term MDR/RR-TB outcomes, such as post-TB lung disease, to refine management strategies tailored to the reality of low-resource, high-burden settings.

## Author summary

Despite improvements in tuberculosis (TB) treatment protocols, multi-drug-resistant and rifampicin-resistant (MDR/RR)-TB outcomes remain suboptimal. Nontuberculous mycobacteria (NTM), environmental organisms opportunistically capable of causing lung disease in susceptible individuals, are increasingly identified from sputum specimens in regions with high TB prevalence. Yet, their clinical impact on MDR/RR-TB outcomes is not well understood. Pulmonary NTM disease often presents with symptoms similar to pulmonary TB, complicating diagnosis and potentially leading to treatment modifications that may not always be warranted.

In Ghana, where MDR/RR-TB cases are on the rise, standardised short-course injectable (SCI) and all-oral bedaquiline-based (SCO) treatment regimens have been in place. However, there is no data on how the presence of NTM may influence MDR/RR-TB treatment response, particularly within different geographic and environmental contexts. There have, however, been reports of NTM emergence in people undergoing treatment for MDR/RR-TB using a bedaquiline-containing regimen in China. We present this gap in the management of MDR/RR-TB, noting the clinical implications of NTM isolation during the course of TB treatment is not well-defined. This study sought to fill existing knowledge gaps by identifying predictors of NTM isolation in sputum samples from patients undergoing treatment for MDR/RR-TB using either the SCO or SCI. Additionally, we evaluated whether the presence of NTM was associated with unfavourable TB treatment outcomes. Our findings indicate geographic differences in the occurrence of NTM among the study cohort. Also, persons living with HIV were more likely to experience unfavourable TB treatment outcomes. The presence of NTM did not affect the MDR/RR-TB treatment outcomes, regardless of the regimen used in our study. Our findings suggest that routine detection of NTM in MDR/RR-TB patients may not necessitate treatment changes. However, the detection of NTM shows the need for further investigation into how regional characteristics may influence NTM coinfection and potentially NTM pulmonary disease. Addressing this challenge is essential for guiding treatment decisions in resource-limited settings.

## Introduction

Multi-drug-resistant and rifampicin-resistant tuberculosis (MDR/RR-TB) remains an important public health challenge globally, including in Sub-Saharan Africa (SSA). In 2023, the World Health Organization (WHO) reported 189 000 laboratory-confirmed cases of MDR/RR-TB worldwide, of which 23 000 were from the WHO African region [1]. In Ghana, case detection increased from 72 in 2015–227 in 2023 [1,2]. A study by Musa et al. found a pooled prevalence of MDR/RR-TB of 2% among new TB cases in SSA [3]. Known factors contributing to unfavourable treatment outcomes in MDR/RR-TB cases include HIV coinfection and previous TB treatment [4,5].

Nontuberculous mycobacteria (NTM) are environmental mycobacteria, of which some are capable of causing disease in humans [6]. *Mycobacterium avium complex* (MAC) and *M. abscessus complex* (MAB) are the commonest NTM species isolated from clinical specimens globally [7]. Vulnerable individuals, such as those with structural lung disease, for instance as seen following TB disease, is the commonest predisposing condition besides ageing and immunosuppressant use [8–10]. Based solely on symptoms, radiological features and smear microscopy, pulmonary infections caused by NTM can be clinically indistinguishable from those caused by the *M. tuberculosis* complex (MTBc), making diagnosis challenging [10]. When clinically significant, NTM lung disease usually follows an indolent disease course [11]. If a decision is made to treat, therapy consists of a multidrug regimen, often containing a macrolide and/or aminoglycoside, over 12 months duration [12,13].

In Canada, NTM were identified in 11% (40/369) of individuals with culture-positive pulmonary TB undergoing treatment, while in Taiwan, NTM were isolated in 7.2% (154/2133) of persons receiving treatment for TB [14,15]. Despite the growing number of studies, including the systematic review by Okoi C. et al. in 2019, which reported an increasing prevalence of NTM in respiratory tract samples of patients with presumed TB in SSA, the potential effect on treatment outcomes of NTM presence in the cultures of patients with pulmonary MDR/RR-TB has not yet been evaluated in SSA [16–18].

Over the past six years, WHO recommended standardised chemotherapy for MDR/RR-TB has evolved significantly [19]. The treatment has shifted from the reliance on fluoroquinolones and second-line injectables, such as kanamycin, capreomycin, and amikacin, to newer agents like bedaquiline, pretomanid and delamanid [20]. The susceptibility of various NTM species to these drugs varies considerably. For instance, the susceptibility of MAC, MAB and *M. fortuitum* to amikacin and kanamycin has been well established, with susceptibility rates ranging between 87–100% [21,22]. Bedaquiline has demonstrated *in vitro* activity against several NTM species, including MAC, MAB, *M. fortuitum*, and *M. kansasii*, especially in extrapulmonary infections. However, its use in pulmonary NTM disease remains limited [23]. While fluoroquinolones, particularly levofloxacin and moxifloxacin, are a cornerstone in the treatment of MDR/RR-TB, their efficacy against most NTM species is limited, except for *M. fortuitum*, which shows proven susceptibility to these drugs [22,23]. This variation in drug susceptibility highlights the complexities involved in treating NTM infections in the context of MDR/RR-TB therapy.

In Ghana, the MDR/RR-TB treatment outcome has been suboptimal from 2018 to 2021, with a success rate ranging from 59% to 68%, despite ongoing interventions. These interventions have included the provision of free MDR/RR-TB treatment, as well as social support measures such as monetary assistance for transportation and investigations, and the supply of food [24–26]. During this period, increasing detection of NTM in mycobacterial cultures was observed, as reported by Abbew et al., with proportions ranging from 6% to 35% of all positive mycobacterial cultures from the National TB Reference Laboratory [27]. Hence, we evaluated predictors of NTM isolation in MDR/RR TB patients, and assessed the effect of NTM presence on treatment outcome, adjusted for potential confounders.

## Methods

### Study design, patient population and setting

This study is a retrospective cohort analysis of patients with MDR/RR-TB in Ghana, conducted between 2018 and 2021. The study period was selected to align with the implementation of the 9–11 month SCI (also known as the Bangladesh regimen: kanamycin, fluoroquinolones, protionamide, high-dose isoniazid, clofazimine, pyrazinamide, and ethambutol) and the SCO (including bedaquiline, levofloxacin, protionamide, high-dose isoniazid, clofazimine, pyrazinamide, and ethambutol). Patients recruited in 2018 and early 2019 were placed on the SCI regimen, while those from late 2019–2021 were on the SCO regimen.

Patients diagnosed with MDR/RR-TB across four regions in Ghana — Ashanti, Eastern, Brong Ahafo, and Greater Accra — were included in this study. We excluded patients with unknown treatment outcomes (patients in whom treatment outcome was not documented) or having additional resistance to the injectables and/or fluoroquinolones. Additionally, patients on a long-course regimen, those with individualised regimens, or those with unknown regimen types were excluded from the final analysis. The study’s objective was to analyse patients with MDR/RR-TB who had no additional resistance, such as to fluoroquinolones, and were receiving the standardised SCI or SCO regimen as described in this session. These regions were selected based on previous evidence from the laboratory analysis conducted by Abbew et al., which reported increasing isolation of NTM species in Ghana between 2012 and 2021, with regional variations in the prevalence of NTM [27].

Our study was embedded in National Tuberculosis Programme (NTP) activities in Ghana, where (RR-)TB diagnosis relies on the Xpert MTB/RIF assay. For patients who lacked direct access to Xpert MTB/RIF facilities, sputum samples were transported to designated sites equipped with this capability. Routine mycobacterial culture and phenotypic drug-susceptibility testing (DST) were conducted at baseline for all individuals diagnosed with MDR/RR-TB. Subsequently, cultures were performed monthly throughout the treatment period to monitor treatment response.

For all positive cultures where MTBc was not identified by the MPT64 Assay (SD Bioline, Seoul, South Korea) and thus presumed to be NTM, species identification was conducted using line probe assays (LPAs) GenoType CM/AS (Bruker, Germany). In case identification to the species level was not possible further analysis using Sanger Sequencing of the *rrs* and or *rpoB* gene at the Institute of Tropical Medicine, Antwerp, Belgium was done for this study.

### Study variables and data sources

Patient-level data from pulmonary MDR/RR-TB patients were collected from medical records at each reporting facility within the study regions. These records provided detailed information on the demographics and clinical characteristics of the patients, including age, sex, HIV status, presence of diabetes mellitus, previous TB history and baseline diagnostic results such as smear microscopy, Xpert MTB/RIF assay, LPA, and chest X-ray findings (categorised as cavitary, consolidation, effusion). The type of treatment regimen administered was also recorded, including whether patients were placed on the standardised short-course injectable (SCI) and all-oral bedaquiline-based (SCO) and the specific drug combinations used in each case.

Laboratory data, including culture and LPA results, were sourced from the database of the TB Reference Laboratory at the Eastern Regional Hospital. Treatment outcomes were categorised based on the national DR-TB treatment guidelines. A favourable outcome was defined as either cured or treatment completed, while an unfavourable outcome included death, lost-to-follow-up, referral, treatment failure, or treatment interruption. Additionally, death and treatment failure were considered clinically adverse outcomes.

### Statistical analysis

All data were compiled from separate spreadsheets from each region and subsequently merged into a single dataset for analysis. For ease of analysis, patients with at least one NTM isolate were categorised as the “NTM” group, while those without any NTM isolates were classified as “no NTM.” Additionally, patients with MDR/RR-TB were further subcategorised into three groups—MDR-TB, RR-TB, and RR-TB with unknown isoniazid (INH) resistance—to evaluate the potential effect of INH resistance on treatment outcomes.

Predictors of NTM isolation were analysed using a multivariable (MV) logistic regression model. A sensitivity analysis was performed to examine the results for patients with NTM species isolated at any time point during the treatment while excluding those with baseline NTM isolated. The association between NTM isolation and having an unfavourable treatment outcome was also assessed using MV logistic regression, adjusting for the type of treatment regimen (SCO and SCI) and other potential confounders. A second sensitivity analysis was conducted to assess predictors of clinically unfavourable outcomes, after excluding patients who were lost to follow-up. The MV models incorporated all variables that were significantly associated with the outcome in the bivariable analysis, along with the variables of interest. In the MV model for predicting NTM isolation, the treatment regimen was included as a variable of interest. For the treatment outcome prediction model, both the regimen type and the presence of NTM were added to the MV model as variables of interest. All statistical analyses were conducted using Stata version 18 (StataCorp, Texas, USA).

### Ethical approval

Ethical approval for this study was obtained from the Institutional Review Board of the Institute of Tropical Medicine (1635/22) and the Ethics Review Committee of the Cape Coast Teaching Hospital (CCTHERC/EC/2022/157). Additionally, permission was granted by the Ghana Health Service through the NTP to access relevant patient data. The study involved retrospective data collection without direct patient contact, and an informed consent waiver was granted. However, it is essential to note that all patients undergoing DR-TB management within the NTP framework are requested to provide informed consent for treatment, follow-up and future research on routinely collected data before treatment initiation.

## Results

### Patient identification and analysis

**Fig 1:**
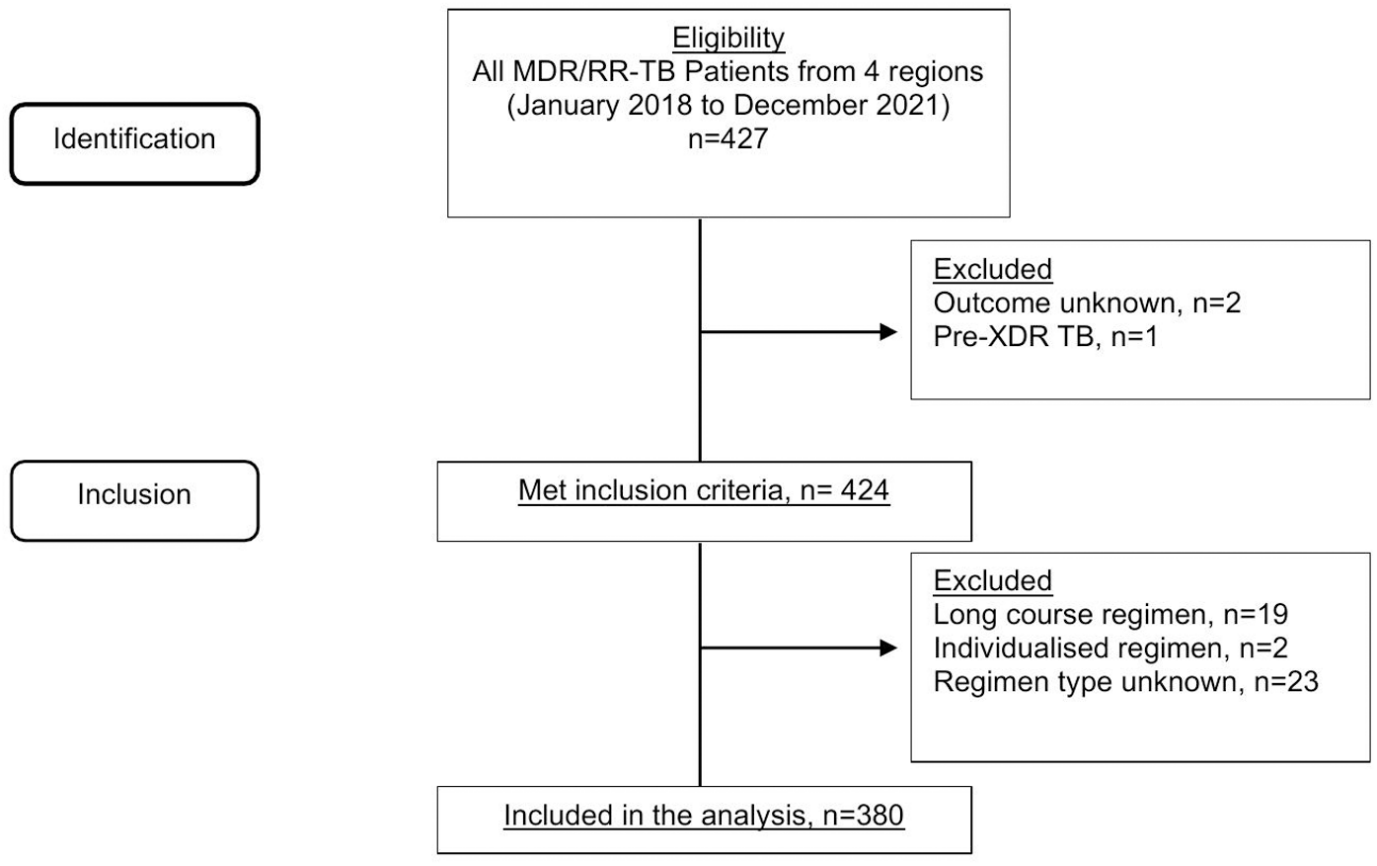
Patient identification and inclusion.

A total of 427 patients with MDR/RR-TB were identified from four regions in Ghana. After excluding patients with unknown treatment outcomes or regimen, those who were identified with resistant to fluoroquinolone and/or injectable drugs, and those treated with a long-course or individualised treatment regimen, 380 patients were included in the analysis (Fig 1).

### Sociodemographic characteristics

Of the 380 patients, the majority were male, comprising 76.3% of the cohort. The mean age of the patients was 43.9 years, and 72 (18.9%) patients were living with HIV (Table 1).

**Table 1:** Sociodemographic characteristics of the patient population.

*Characteristics*	*Total (N = 380)*		*Population without NTM* *(N = 353)*		*Population with NTM (N = 27)*		*P-value (Chi2)*
	*n*	*(%)*	*n*	*(%)*	*n*	*(%)*	
Age (years), mean, SD	43.9,	15.0	43.9, 15.0		43.7,	16.1	
Age groups							0.90
0-19	12	(3.2)	11	(3.1)	1	(3.7)	
20-39	138	(36.3)	127	(36.0)	11	(40.7)	
40-59	170	(44.7)	158	(44.8)	12	(44.4)	
>60	60	(15.8)	57	(16.1)	3	(11.1)	
Sex							0.85
Male	290	(76.3)	269	(76.2)	21	(77.8)	
Female	90	(23.7)	84	(23.8)	6	(22.2)	
Region							<0.001
Greater Accra	85	(22.4)	84	(23.8)	1	(3.7)	
Ashanti	162	(42.6)	158	(44.8)	3	(11.1)	
Eastern	81	(21.3)	64	(18.1)	18	(66.7)	
Bono Ahafo	52	(13.7)	47	(13.3)	5	(18.5)	
DR-TB Type							0.01
MDR-TB	219	(57.6)	155	(43.9)	6	(22.2)	
RR-TB	161	(42.4)	189	(53.5)	18	(66.7)	
RR-TB with INH DST unknown	12	(3.2)	9	(2.5)	3	(11.1)	
HIV Status							0.38
Positive	72	(18.9)	69	(19.6)	3	(11.1)	
Negative	300	(78.9)	276	(78.2)	24	(88.9)	
Unknown	8	(2.1)	8	(2.3)	0	(0.0)	
Type of patient							0.02
New	199	(52.4)	191	(54.1)	8	(29.6)	
Previous TB history *Of which*	181	(47.6)	151	(42.8)	17	(63.0)	
Relapse	108	(28.4)	99	(28.0)	9	(33.3)	
Treatment after failure	27	(7.1)	23	(6.5)	4	(14.8)	
Treatment after loss to follow-up	23	(6.1)	20	(5.7)	3	(11.1)	
Other previously treated	10	(2.6)	9	(2.5)	1	(3.7)	
TB treatment history is unknown	13	(3.4)	11	(3.1)	2	(7.4)	
Regimen type							0.53
All-oral bedaquiline regimen	260	(68.4)	238	(67.4)	21	(77.8)	
Short course injectable-containing regimen	120	(31.6)	115	(32.6)	6	(22.2)	

*Radiological presentation: At baseline, 13 patients had radiological documentation, 11 with consolidation and 2 with cavitations. Both patients with cavitations were in the NTM group.*

*DR-TB = drug resistant tuberculosis; HIV = human immunodeficiency virus; INH = isoniazid; MDR-TB = multi-drug-resistant tuberculosis; NTM = nontuberculous mycobacteria; RR-TB = rifampicin resistant tuberculosis; SD = standard deviation; TB = tuberculosis DST = Drug susceptibility testing.*

### Clinical characteristics and predictors of NTM isolation

Among the 380 patients in the study, 27 (7.1%) had NTM isolated at some point during their treatment (Table 2). Among these, six patients had NTM isolated at baseline; one of whom subsequently had a different NTM species isolated at month 8, while the remaining five had no NTM isolated during treatment follow up (Table 2). Among the 21 patients with no documented NTM isolation at baseline, three patients had multiple positive NTM isolates during the course of treatment, with two of them having repeated isolates of the same NTM species *(M. Intracellulare)*. Overall, the most common NTM species were *M. intracellulare* (21/27, 77.8%) and *M. fortuitum* (4/27, 14.8%). On average, patients included in the study had on average four cultures conducted from diagnosis to the end of treatment. The median time of isolation was month 5 of treatment (interquartile range [IQR]: 4–8 months). Among the 27 patients with NTM isolation, 21 (77.8%) were male, and 17 (63.0%) had previous TB treatment.

**Table 2:** Individual patient characteristics of patients identified with NTM, stratified by NTM isolation and treatment outcome.

Demographics							Baseline				During follow up	
Age, Sex	Region	BMI (kg/m2)	RR/MDR TB	Regimen Type	Previous TB	HIV Status	Chest X-ray at baseline	Xpert at baseline	Smear at baseline	Species at baseline	NTM species (timing)	Outcome of MDR/RR- TB
44, M	Eastern	19.8	RR-TB	SCO	Yes	Positive (TDF + 3TC + EFV)	Not retrieved	MTB Detected very low	Positive	*M. fortuitum*	*M. intracellulare* (month 8)	Cured
45, M	Eastern	NA	RR-TB	SCO	Yes	Negative	Consolidation	MTB Detected High	Positive	*M. intracellulare*	none	Cured
25, M	BA	20.2	RR-TB	SCO	Yes	Negative	Not retrieved	MTB Detected very low	Positive	*M. intracellulare*	none	Cured
36, M	Ashanti	22.9	RR-TB	SCO	Yes	Negative	Consolidation	MTB Detected High	Positive	*M. intracellulare*	none	Cured
55, M	GAR	21.4	RR-TB	SCO	Yes	Negative	Not retrieved	MTB Detected medium	Positive	*M. intracellulare*	none	Treatment completed
59, M	Eastern	NA	RR-TB	SCO	Yes	Negative	Cavitation with consolidation	MTB Detected low	Positive	*M. abscessus* complex	none	Death
75, F	BA	20.2	RR-TB	SCO	Yes	Negative	Not retrieved	MTB Detected medium	Positive	MTBc	*M. intracellulare* (month 1)	Cured
49, F	Eastern	13.3	RR-TB	SCO	Yes	Negative	Not retrieved	MTB Detected very low	Positive	MTBc	*M. intracellulare* (month 2, 8, 9)	Cured
50, F	Eastern	24.8	RR-TB	SCO	No	Positive (TDF + 3TC + EFV)	Not retrieved	MTB Detected very low	Positive	MTBc	*M. intracellulare* (month 5)	Cured
29, F	Eastern	14.2	RR-TB	SCO	No	Negative	Consolidation	MTB Detected high	Positive	MTBc	*M. intracellulare* (month 5)	Cured
30, M	Eastern	17.3	MDR-TB	SCI	No	Negative	Consolidation	MTB Detected medium	Unknown	MTBc	*M. intracellulare* (month 6)	Cured
45, M	Eastern	16.6	RR-TB	SCO	Yes	Negative	Not retrieved	MTB Detected medium	Positive	MTBc	*M. intracellulare* (month 9)	Cured
37, M	BA	21.8	MDR-TB	SCI	Yes	Negative	Not retrieved	MTB Detected medium	Positive	MTBc	*M. intracellulare* (month 4)	Treatment completed
31, M	BA	20.8	MDR-TB	SCI	No	Negative	Consolidation	MTB Detected trace	Positive	MTBc	*M. intracellulare* (month 8)	Treatment completed
36, M	BA	20	RR-TB	SCO	Yes	Negative	Not retrieved	MTB Detected medium	Positive	MTBc	*M. intracellulare* (month 6)	Treatment completed
41, M	Eastern	15	MDR-TB	SCI	Yes	Negative	Not retrieved	Unknown	Positive	MTBc	*M. intracellulare* (month 5)	Death
9, M	Eastern	14	RR-TB	SCO	Yes	Positive	Not retrieved	MTB Detected medium	Positive	MTBc	*M. intracellulare* (month 3)	Death
82, F	Eastern	19.6	MDR-TB	SCI	No	Negative	Not retrieved	MTB Detected very low	No data	No data	*M. fortuitum* (month 4)	Cured
32, F	Eastern	13.9	RR-TB	SCO	Yes	Negative	Consolidation	MTB Detected very low	No data	No data	*M. intracellulare* (month 4)	Cured
47, M	Ashanti	20	RR-TB	SCO	Yes	Negative	Not retrieved	MTB Detected medium	No data	No data	*M. fortuitum* (month 8)	Cured
58, M	Ashanti	20.3	MDR-TB	SCI	Yes	Negative	Not retrieved	MTB Detected medium	Positive	No data	*M. malmoense* (month 8)	Cured
29, M	Eastern	19.3	RR-TB	SCO	Yes	Negative	Consolidation	MTB Detected very low	No data	No data	*M. intracellulare* (month 8, 9)	Cured
64, M	Eastern	NA	RR-TB	SCO	No	Negative	Not retrieved	MTB Detected medium	No data	No data	*M. intracellulare* (month 12)	Cured
30, M	Eastern	NA	MDR-TB	SCO	Unknown	Negative	Not retrieved	MTB Detected medium	No data	No data	*M. intracellulare* (month 5)	Treatment completed
56, M	Eastern	NA	RR-TB	SCO	Yes	Negative	Not retrieved	MTB Detected medium	No data	No data	*M. fortuitum* (month 3)	Lost to follow-up
37, M	Eastern	NA	RR-TB	SCO	No	Negative	Not retrieved	MTB Detected medium	No data	No data	*M. intracellulare* (month 6)	Lost To Follow-Up
54, M	Eastern	27.5	RR-TB	SCO	No	Negative	Consolidation and cavitation	MTB Detected medium	Negative	Negative culture	*M. intracellulare* (month 4) *M. avium* (month 6)	Cured

*BA = Brong Ahafo region; 3TC = lamivudine; TDF = tenofovir; EFV = efavirenz; GAR = Greater Accra Region; MDR-TB = multi-drug-resistant tuberculosis; MTB = Mycobacterium tuberculosis; NTM = nontuberculous mycobacteria; RR-TB = rifampicin resistant tuberculosis; SCI = short course injectable-containing regimen; SCO = short course all-oral regimen; TB = tuberculosis, All Previously Treated patients were given category 1 treatment comprising rifampicin, isoniazid, ethambutol, pyrazinamide for two months followed by rifampicin, isoniazid for four months in their initial treatment course.*

Patients with previous TB treatment had higher odds of NTM isolation during their treatment in bivariable analysis (odds ratio [OR] 2.68, 95% confidence interval [CI]: 1.19-7.38), but this was no longer statistically significant in multivariable analysis (adjusted odds ratio [aOR] 2.3, 95% CI: 0.84-6.08) (Table 3). Individuals with MDR/RR-TB from the Eastern Region (aOR 15.68, 95% CI: 4.11-59.79) and Brong Ahafo region (aOR 4.74, 95% CI 1.03-21.60) had increased odds of NTM isolation. None of the other clinical or demographic predictors were significantly associated with NTM isolation.

**Table 3:** Predictors of isolating NTM in patients with MDR/RR-TB from 2018-2021 in four regions in Ghana.

	No NTM		NTM					
	N = 353		N = 27		OR	(95% CI)	aOR	(95% CI)
	n	(%)	n	(%)				
**Age groups, N = 380**							NS	
Below 20 years	11	(91.7)	1	(8.3)	Ref			
20 - 39 years	127	(92.0)	11	(8.0)	0.95	(0.11-8.08)		
40 - 59 years	158	(92.9)	12	(7.1)	0.84	(0.09-7.03)		
60 years and above	57	(95.0)	3	(5.0)	0.57	(0.05-6.09)		
**Sex, N = 380**							NS	
Female	84	(93.3)	6	(6.7)	Ref			
Male	269	(92.8)	21	(7.2)	1.09	(0.42-2.79)		
**Region, N = 380**								
Ashanti	158	(98.1)	3	(1.9)	Ref		Ref	
Brong Ahafo	47	(90.4)	5	(9.6)	5.60	(1.29-24.32)	4.74	(1.03-21.60)
Eastern	64	(78.0)	18	(22.0)	14.80	(4.21-52.02)	15.68	(4.11-59.79)
Greater Accra	84	(23.8)	1	(1.2)	0.62	(0.06-6.12)	1.22	(0.11-14.19)
**DR-TB Type, N = 380**								
MDR-TB	155	(95.3)	6	(3.7)	Ref		Ref	
RR-TB	189	(91.3)	18	(8.7)	2.46	(0.95-6.36)	2.34	(0.79-6.93)
RR-TB with INH unknown	9	(75.0)	3	(25.0)	8.61	(1.84-40.17)	3.88	(0.53-28.60)
**Previous TB treatment, N = 369**								
No	191	(96.0)	8	(4.0)	Ref		Ref	
Yes	151	(89.4)	17	(10.6)	2.68	(1.19-7.38)	2.26	(0.84-6.08)
**HIV status, N = 375**							NS	
Negative	276	(92.0)	24	(8.0)	Ref			
Positive	69	(95.8)	3	(4.2)	0.50	(0.14-1.70)		
Unknown	8	(100.0)	0	(0.0)				
**Smear at baseline, N = 380**							NS	
Negative	37	(94.9)	2	(5.1)	Ref			
Positive	186	(92.5)	15	(7.5)	1.49	(0.33-6.80)		
Unknown	130	(92.9)	10	(7.1)		NA		
**BMI (kg/m** ^**2**^ **), N = 326**							NS	
>=18	205	(93.6)	14	(6.4)	Ref			
<18	100	(93.6)	7	(6.5)	1.03	(0.40-2.62)		
**Xpert at Baseline, N = 363**							NS	
Group 1 (high, medium)	235	(94.0)	15	(6.0)	Ref			
Group 2 (low, very low, trace)	104	(92.0)	9	(8.0)	1.36	(0.51-3.43)		
**Regimen, N = 380**								
All-oral bedaquiline	238	(91.9)	21	(8.1)	Ref		Ref	
Short-course injectable	115	(95.0)	6	(5.0)	0.59	(0.23-1.50)	1.35	(0.44-4.13)

*BMI = body mass index; DR-TB = drug-resistant tuberculosis; HIV = human immunodeficiency virus; INH = isoniazid; MDR-TB = multi-drug resistant tuberculosis; NA: Not applicable; NS: Not significant; NTM = nontuberculous mycobacteria; RR-TB = rifampicin-resistant tuberculosis; TB = tuberculosis; OR=odds ratio; aOR=adjusted odds ratio; 95% CI = 95% confidence interval*

In the sensitivity analysis, where NTM isolation was limited to follow-up cultures only, individuals from the Eastern Region (aOR 15.47, 95% CI: 3.38-70.90) and Brong Ahafo Region (aOR 6.28, 95% CI: 1.10-35.64) had a significantly increased odds of NTM isolation.

### Treatment outcomes of MDR/RR-TB

Overall, 67.9% achieved a favourable treatment outcome (Table 4), which was higher in the SCO group (71.4%) compared to the SCI group (60.3%). NTM isolation was not associated with unfavourable MDR/RR-TB treatment outcome. Among the entire cohort of patients, the odds of experiencing an unfavourable treatment outcome was higher for those who were people living with HIV (aOR 1.80, 95% CI: 1.05-3.11).

**Table 4:** Predictors for unfavourable outcome among MDR/RR-TB patients.

	Favourable, N = 258		Unfavourable, N = 122		OR	(95% CI)	aOR	(95% CI)
	n	(%)	n	(%)				
**Age group, N = 380**							NS	
Below 20 years	6	(50.0)	6	(50.0)	Ref			
20 - 39 years	97	(70.3)	41	(29.7)	0.42	(0.13-1.39)		
40 - 59 years	118	(69.4)	52	(30.6)	0.44	(0.14-1.43)		
60 years and above	37	(61.7)	23	(38.3)	0.62	(0.18-2.16)		
**Sex, N = 380**							NS	
Female	62	(68.9)	28	(31.1)	Ref			
Male	196	(67.6)	94	(32.4)	1.06	(0.64-1.77)		
**Region, N = 380**								
Ashanti	105	(65.2)	56	(34.8)	Ref		Ref	
Brong Ahafo	42	(80.8)	10	(19.2)	0.45	(0.21-0.98)	0.58	(0.26-1.29)
Eastern	53	(64.6)	29	(35.4)	1.03	(0.59-1.79)	1.51	(0.80-2.83)
Greater Accra	58	(68.2)	27	(31.8)	0.87	(0.50-1.53)	1.22	(0.63-2.35)
**DR-TB Type, N = 380**							NS	
MDR-TB	114	(70.8)	47	(29.2)	Ref			
RR-TB	139	(67.2)	68	(32.8)	1.18	(0.76-1.85)		
RR-TB with INH unknown	5	(41.7)	7	(58.2)	NA			
**Previous TB treatment, N = 369**							NS	
No	133	(66.8)	66	(33.2)	Ref			
Yes	122	(71.8)	48	(28.2)	0.79	(0.51-1.23)		
**HIV status, N = 380**								
Negative	214	(71.3)	86	(28.7)	Ref		Ref	
Positive	42	(58.3)	30	(41.7)	1.78	(1.04-3.02)	1.80	(1.05-3.11)
Unknown	2	(25.0)	6	(75.0)	NA		NA	
**Smear at baseline, N = 380**							NS	
Negative	29	(74.4)	10	(25.6)	Ref			
Positive	137	(68.2)	64	(31.8)	1.35	(0.62-2.95)		
Unknown	92	(65.7)	48	(34.3)	NA			
**BMI (kg/m** ^**2**^ **), N = 326**							NS	
>=18	158	(72.1)	61	(27.9)	Ref			
<18	75	(70.1)	32	(29.9)	1.11	(0.66-1.84)		
**Regimen Type, N = 380**								
All-oral bedaquiline	185	(71.4)	74	(28.6)	Ref		Ref	
Short-course injectable	73	(60.3)	48	(39.7)	1.68	(1.04-2.59)	1.70	(0.99-2.94)
**NTM isolate, N = 380**								
No NTM	236	(66.9)	117	(33.1)	Ref		Ref	
At least one NTM	22	(81.5)	5	(18.5)	0.46	(0.17-1.25)	0.45	(0.16-1.31)
**Xpert at Baseline, N = 363**							NS	
Group 1 (high, medium)	165	(66.0)	85	(34.0)	Ref			
Group 2 (low, very low, trace)	76	(73.3)	27	(26.2)	0.61	(0.35-1.03)		

*The unknown covariates of HIV status and smear microscopy, as well as RR-TB with INH unknown, were dropped from the analysis*.

*BMI = body mass index; DR-TB = drug-resistant tuberculosis; HIV = human immunodeficiency virus; INH = Isoniazid; MDR-TB = multi-drug resistant tuberculosis; NA: Not applicable; NTM = nontuberculous mycobacteria; NS: Not significant; RR-TB = rifampicin-resistant tuberculosis; TB = tuberculosis; OR=odds ratio; aOR=adjusted odds ratio; 95% CI = 95% confidence interval.*

In a sensitivity analysis, estimating predictors of clinically unfavourable outcomes, HIV-seropositivity was associated with an almost two-fold increased risk of having a clinical adverse outcome (aOR 1.98, 95% CI: 1.01-3.85).

## Discussion

The global prevalence of NTM isolation is rising, with SSA also experiencing a notable increase in many settings [28]. NTM lung disease risks being misdiagnosed as TB in high-TB-incidence settings [29]. Furthermore, studies, such as those by Maiga et al., have identified NTM pulmonary disease (NTM-PD) in individuals initially categorised as having ‘recurrent’ or drug resistant-TB (DR-TB) [30]. Despite these findings, no study to date has evaluated the role of NTM isolation on MDR/RR-TB treatment outcomes or investigated predictors of NTM isolation in patients diagnosed with MDR/RR-TB.

Our study compared the treatment outcomes of MDR/RR-TB patients who had NTM isolated during their treatment course, with a focus on the different regimes utilised, specifically the SCI and SCO. Although the proportion of patients with NTM isolation was numerically higher in the SCO group (8.1%) compared to the SCI group (4.9%), it did not affect treatment outcome (Table 4). With the WHO’s decision in 2022 to discontinue the use of injectable-containing regimens in favour of all-oral regimens, except for amikacin in children and individualised treatment regimens, the landscape of MDR/RR-TB treatment has shifted [31]. However, studies evaluating pulmonary NTM disease in individuals treated with these newer, shorter regimens remain limited globally. As the BPaL(M) (bedaquiline, pretomanid, linezolid, with or without moxifloxacin) regimens are rolled out and more NTM isolation is reported in patients with presumed TB [18,32], there is a critical need for more longitudinal studies with larger sample sizes to assess the long-term impact of NTM on MDR/RR TB treatment outcomes.

As our findings show that NTM isolation did not affect treatment outcome in this cohort, treatment modification for MDR/RR-TB may not be necessary solely based on the isolation of NTM. However, it emphasises the need for careful clinical monitoring to distinguish between transient NTM colonisation, environmental sample contamination and NTM-related lung disease, which may warrant treatment adjustments. NTM are environmental organisms, commonly isolated from water (plumbing) systems [33]. With the increasing detection of NTM species in the sputum of patients diagnosed with TB, it is crucial to consider the potential role of environmental contamination of laboratory samples in contributing to NTM isolation.

*M. intracellulare* and *M. fortuitum* were the commonest isolated species in our cohort, which is in line with regional data on predominant MAC. However, *M. scrofulaceum,* which was not identified in our study, has been shown to be more prevalent than *M. fortuitum* in SSA [18,34].

Prior TB has been identified as a significant risk factor for NTM-PD in previous studies [18,35]. In our study, previous TB treatment was not associated with increased risk of NTM isolation, which may be related to the small sample size. Furthermore, we studied NTM isolation in an MDR/RR-TB patient cohort with very few of them fulfilling ATS/IDSA diagnostic criteria for NTM-PD. Notably, 21 out of 27 patients did not present with baseline NTM, yet subsequently exhibited NTM isolation following several months of treatment for MDR/RR-TB. Hence, we were unable to assess the clinical relevance of NTM isolation during short course MDR/RR-TB therapy in our cohort given the indolent nature of NTM infection/disease, lack of long-term follow-up and the possible environmental presence of NTM [33].

A geographical variation was evident, with patients from the Eastern and Brong Ahafo regions having notably higher odds of NTM isolation. While mining activities are widespread throughout Ghana, the increased NTM isolation in these regions suggests that local environmental factors may play a role in NTM acquisition in the airways. The contribution of climate change and occupational exposure is well known [36,37], but data from SSA are lacking. The Eastern and Brong Ahafo regions, characterised by a more verdant agricultural landscape, are home to many farming communities, and further investigation is warranted to better understand the epidemiological patterns of NTM observed in these areas.

Our findings suggest that the isolation of NTM does not appear to have an effect on treatment outcomes of MDR/RR-TB patients. This indicates that treatment modifications may not be necessary based solely on the presence of NTM. However, given the small sample size and potential environmental contamination, further large-scale, longitudinal investigations are needed to elucidate the clinical relevance of NTM isolation in the management of MDR/RR-TB.

## Strengths, limitations and conclusion

This study provides the first documented data on the impact of NTM isolation on MDR/RR-TB outcomes in a TB-endemic setting in SSA with a comparison between two regimens. These findings are particularly relevant for low-resource, high-TB burden regions, where evidence of NTM co-infection in MDR/RR-TB is scarce and may influence management strategies. However, this study also has some limitations. First, the small sample size, coupled with missing data, particularly regarding radiological findings, limits the strength of the conclusions. The clinical relevance of a single NTM isolate remains uncertain in the context of TB lung disease. While NTM are often environmental pathogens, their role in causing clinically significant disease versus being mere colonisers remains unclear, especially in TB-endemic regions where TB remains a priority. More long-term follow-up studies are required to clarify the pathogenic significance of NTM in patients with a history of TB.

## Supporting information

S1 File Dataset underlying study analysis. (XLSX)
